# The regulatory effects of *Pseudostellaria heterophylla* polysaccharide on immune function and gut flora in immunosuppressed mice

**DOI:** 10.1002/fsn3.2979

**Published:** 2022-08-24

**Authors:** Yongjun Kan, Yingying Liu, Yating Huang, Li Zhao, Chang Jiang, Yanjin Zhu, Ziqin Pang, Juan Hu, Wensheng Pang, Wenxiong Lin

**Affiliations:** ^1^ Fujian Agriculture and Forestry University Fuzhou China; ^2^ Fujian Academy of Chinese Medical Sciences Fuzhou China; ^3^ The Second Affiliated Hospital of Fujian University of Traditional Chinese Medicine Fuzhou China; ^4^ Fujian University of Traditional Chinese Medicine Fuzhou China

**Keywords:** gut flora, immunomodulatory activity, immunosuppressed, polysaccharides, *Pseudostellaria heterophylla*

## Abstract

Polysaccharide (PF40) has been recognized as a main bioactive substances in *Pseudostellaria heterophylla* (Miq.). The current study explored the potential protective effects of PF40 on immune system in mice with cyclophosphamide‐induced immunosuppression. The mice were intragastric administered PF40 at the dosage of 100, 200 or 400 mg/kg once daily for 30 days, On the 24th and 25th day, the additional intraperitoneal injection of PF40 (50 mg/kg) were administered. The results showed that PF40 enhanced the cell‐mediated immunity via improvements in macrophage phagocytosis, splenocyte proliferation, NK cell activity and delayed type hypersensitivity. Equally, it improves humoral immunity through promoting the formation of serum hemolysin. Moreover, PF40 maintain the immune balance of splenic lymphocytes and altered the intestinal physiological status in Cyp‐induced mice. PF40 regulates the intestinal microbiota by restoring the relative abundance of *Odoribacter* and *Mucispirillum* and reducing the relative abundance of *Sporosarcina*, *Yaniella*, and *Jeotgalicoccus* in Cyp‐intervened mice. The findings suggested that PF40 might be a promising natural functional foods for reducing chemotherapy‐induced immunosuppression.

## Graphical abstract

We discovered for the first time that the polysaccharides of *Pseudostellaria heterophylla* can significantly enhance the immune function of immunosuppressed mice. *Pseudostellaria heterophylla* polysaccharide might be a promising natural functional foods for reducing chemotherapy‐induced immunosuppression.

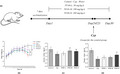

## 1 INTRODUCTION

Cyclophosphamide (Cyp), as a kind of chemotherapeutic drug, has been commonly used for anticancer treatment. However, many studies (Superfin et al., 2007; Tang et al., 2018) have reported that the side effects caused by Cyp, such as multiple organ dysfunction and immunosuppression can significantly limit the use of Cyp in clinical practice. It is necessary to take drugs or food supplements with immunomodulatory function at the same time. Discovery of new drugs with immunostimulatory and detoxification properties would shoulder a mighty significance to improve the patients’ quality of life undergoing chemotherapy.

Natural polysaccharides are widely existed in plants, microorganisms and animals, and are often used as biological response modifiers (Gangalla et al., 2021; Park et al., 2020). In recent years, it has been reported that the polysaccharides extracted from medicinal plants can affect the immune system of body both in vivo and in vitro. Polysaccharides with negligible by‐effect and low level of toxicity are often chosen as optimal immunomodulatory agents. *Ligustrum vicaryi* polysaccharides can effectively improve the indexes of spleen and thymus, enhance the phagocytic function of neutrophils, promote immunomodulatory activity by activating adaptive and innate immunity (Liu et al., 2020). Edible Fungal polysaccharides can effectively inhibit the reduction of IL‐10 levels in mice serum caused by cyclophosphamide and enhance the phagocytosis of mouse peritoneal macrophages (Zhang et al., 2017).

*Pseudostellaria heterophylla* (Miq.) Pax ex Pax et Hoffm, which is also known as Tai‐Zi‐Shen (TZS) has been viewed as a kind of “qi‐enriching and tonic drug.” TZS was regarded as one precious medicine in ancient China, but it is now commonly used in clinical practice. According to previous studies, the main components of *P. heterophylla* are polysaccharides, volatile oily substances, amino acids, saponins, and cyclic peptides (Hu et al., 2019; Pang et al., 2011; Zhao et al., 2022). TZS is widely used in the prescription of cancer patients after chemotherapy. Polysaccharides are considered to be the effective ingredient of TZS. Recently, it has attracted much attention due to its medicinal value, for example, immunomodulation and antitumor (Wong et al., 1994; Yang et al., 2019, 2020).

Our previous series of studies on polysaccharides of TZS found that the PF40 component is the main active component of crude polysaccharides of *P. heterophylla*. The biological activity of PF40 includes increasing insulin secretion, improving insulin resistance, increasing glucose uptake and utilization in cells of muscle and adipose, reducing blood sugar and serum total triglyceride levels, inhibiting the expression of TNF‐α and increasing IL‐10 levels (Chen et al., 2018; Chen, Pang, et al., 2016; Hu et al., 2013) etc. However, the modulating effect of PF40 on the immune response has not been reported yet. Therefore, a study is needed to explore the immunomodulatory activity of PF40.

This current study was therefore conducted to explore the protective effects of PF40 on immune system in Cyp‐induced immunosuppressed BALB/c mice. This study provides evidence in terms of developing PF40 as an alternative medicine for immune protection in cancer patients after chemotherapy from a theoretical perspective.

## 2 MATERIALS AND METHODS

### 2.1 Materials and reagents

*Pseudostellaria heterophylla* was provided from GAP base in Zherong County, Fujian Province, China. PF40 was prepared as per a previous study (Hu et al., 2013). Injectable cyclophosphamide (Cyp) used in this study was purchased from Baxter Oncology GmbH. Concanavalin A (ConA), lipopolysaccharide (LPS, from Escherichia coli O111:B4) and Cell Counting Kit‐8 (CCK‐8) were from Sigma‐Aldrich. RPMI‐1640 medium, streptomycin, fetal bovine serum (heat‐inactived), penicillin, and phosphate buffer saline, were purchased from Gibco Life Technologies. Erythrocyte lysis buffer was from Beyotime Biotechnology. Mouse anti‐CD3‐PE, mouse anti‐CD8‐APC, mouse anti‐CD4‐FITC, and staining buffer came from Multi Science. Mouse ELISA kits (TNF‐α and IFN‐γ) were from eBioscience. Other reagents used in this study were all provided by commercial suppliers without any treatments, unless otherwise noted.

### 2.2 Animal welfare and ethical statements

Inbred strain male Balb/c mice (6–7 weeks old, 22 ± 2 g, specific‐pathogen‐free grade) were purchased from the SLAC Laboratory Animal Co., Ltd. (License ID: SCXK [Shanghai] 2017‐0005) in Shanghai. All animals were housed in a comfortable environment (12 h light–dark cycle, 20 ± 2°C with the humidity of 50 ± 10%) and were fed with rodent chow and water ad libitum. We recorded the weight of mice every week. Animal experiments of this study were granted by the Institutional Animal Care and Use Committee at Fujian Academy of Chinese Medical Sciences (no. FJATCM‐IAEC2021007). All study procedures were conducted by following the Guide for the Care and Use of Laboratory Animals (China).

### 2.3 Experimental design of animals

**FIGURE 1:**
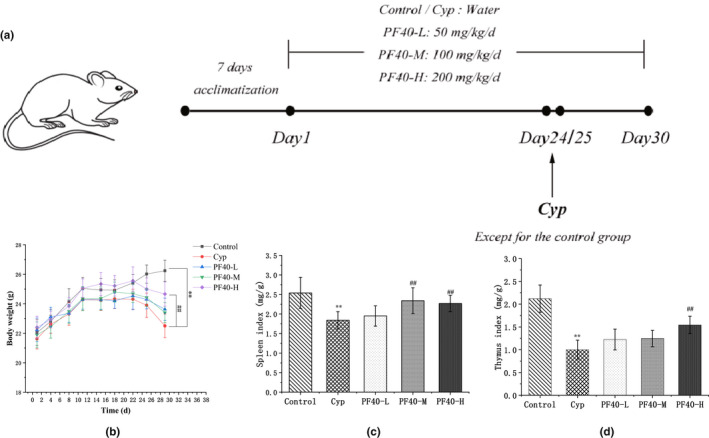
Effect of PF40 on bodyweight and immune organ index in Cyp‐induced immunosuppressed mice (mean ± SD, *n* = 10). (a) Protocol of the animal experiment in this study; (b) Changes of body weight of mice; (c) Spleen index; and (d) Thymus index. **p < .01 versus the control group; ##*p* < .01 versus the Cyp‐treated group.

Protocol of the animal experiment is shown in Figure 1a. A total of 200 mice were involved, which were firstly allocated into four batches (50 mice in each). The 50 mice in each batch were then randomly assigned into the following five groups: the normal control group (Control), Cyp‐induced immunosuppression model group (Cyp), PF40 low‐dose group (PF40‐L), PF40 Medium‐dose group (PF40‐M), and PF40 high‐dose group (PF40‐H). All the mice were housed in the laboratory for 1 week to acclimatize to the laboratory environment. The mice assigned in normal control group and Cyp‐induced model group were fed with distilled water for 1 month (30 days). In the PF40‐L, PF40‐M, and PF40‐H groups, the mice were administered with 100, 200, and 400 mg/kg/day of PF40 by intragastric gavage. On day 24, all the mice in model group, PF40‐L, PF40‐M, and PF40‐H groups were given Cyp intraperitoneally once per day with the dosage of 50 mg/kg for two consecutive days, while the mice in normal control group was treated with a same dose of saline.

### 2.4 Carbon clearance assay

The method of carbon clearance was employed to assess the phagocytic activity of the reticuloendothelial system (Esseddik et al., 2020). This assay was conducted based on previous studies (Mbaoji et al., 2020) with minor modifications. Two hours after the final intragastric administration, the weight of the 10 mice from each group were measured before injecting the Indian ink (0.1 ml/10 g BW, diluted with Normal saline at 1:8 dilution) through the tail vein. At 2 and 10 min immediately after the injection of carbon ink, a 20 μl blood was extracted from the retro‐orbital sinus with mild ether anesthesia. All blood samples were mixed with 2 ml of w/v Na_2_CO_3_ solution (0.1% w/v), and the absorbance was examined at 660 nm. Mice were subsequently sacrificed via the approach of cervical dislocation. Afterwards, the liver and spleen of mice were immediately isolated and weighed. Following is the formula for calculating the rate of carbon clearance (*K*) and the phagocytic index (*α*):

$$K=\frac{\mathrm{lg}A_{1}-\mathrm{lg}A_{2}}{8}\;\left(A1:\text{the absorbance}\;\mathrm{at}\;2\;\min;A2:\text{the absorbance}\;\mathrm{at}\;10\;\min\right)$$

α=K13×Body weight/Weight of liver+Weight of spleen.

### 2.5 Delayed type hypersensitivity

On day 25, to sensitize the mice, 50 μl of 1% DNFB in mixed acetone–olive oil (4:1, v/v) was used on the abdomen skin of mice (depilated with depilatory creams). On day nine, all mice contacted hypersensitivity by using 10 μl of 1% DNFB on both sides of their right ears, 1 h after the last dose; the left ear of mice was handled with olive oil only. After 24 h, all the mice were sacrificed via the approach of cervical dislocation; Afterwards, a puncher was used to take a piece of ear with a diameter of 8 mm from each earlap and then weighed. Weight difference between the right and left ears was calculated to assess ear swelling of the mice. The calculation formula was.

$\mathrm{Ear}\;\text{swelling rate}\;\left(\%\right)=\left(\text{Weight of right}\;\mathrm{ear}-\text{Weight of left}\;\mathrm{ear}\right)/\text{Weight of left}\;\mathrm{ear}.$

### 2.6 Measurement of serum hemolysin

Half‐maximal hemolysin antibody concentration (HC_50_) has been used as a parameter to reflect the level of serum hemolysin. In this study, the method recommended by Huang et al. (2017) was used to determine the hemolysin level in serum. On the 25th day of the experiment, to induce immunological reaction of the mice, 10 mice from each of the study group were immunized with 2% sheep red blood cells (SRBC) via an intraperitoneal injection approach. Five days later, the blood sample of mice was collected from eye orbit; all the samples were clotted for 1 h before centrifuging at 1000 *g* for 10 min to get the serum. The serum was diluted with the SA buffer with the ratio of 1:100. An aliquot of 50 μl of SRBCs (10%, v/v) was mixed with 100 μl of fresh guinea pig serum and 100 μl of diluted serum (diluted with the above mentioned SA buffer at a ratio of 1:8). After 30 min incubation at the temperature of 37°C, to remove intact erythrocytes, we immediately placed the reaction mixture on an ice bath and then centrifuged at 1000 *g* for 10 min. Afterwards, a 50 μl of supernatant was transferred into a 96‐well plate with150 μl of Dush's solution (K_3_Fe [CN]_6_ 0.2 g, NaHCO_3_ 1.0 g, and KCN 0.05 g into 1000 ml distilled water). Meanwhile, we mixed 12.5 μl of 10% (v/v) SRBCs with 200 μl of Dush's solution. After 10 min, the absorbance was measured at 540 nm. The level of serum hemolysin was measured with the half value of hemolysis (HC_50_): HC_50_ = OD_1_/OD_2_ × dilution folds of serum (OD_1_: the absorbance of the sample; OD_2_: the absorbance of SRBCs).

### 2.7 Isolation of splenic lymphocytes

On the 30th day, once all the treatments completed, the approach of cervical dislocation was used to kill the BALB/c mice in each group. All the mice were then disinfected in 75% ethanol for 3 min. Spleens were aseptically dissected from the sacrificed mice; Spleens were minced and gently squeezed in cold RPMI‐1640 medium, then the single‐cell suspension was obtained via using a 70‐μm‐pore sieve mesh. The suspension was washed two times with phosphate‐buffered saline and centrifuged at 450 *g* for 10 min with the temperature of 4°C. The isolated erythrocytes in the cell pellets were lysed using Red Blood Cell Lysing Buffer, it was then washed for two times with RPMI‐1640 after an incubation of 10 min. Afterwards, the final cells were resuspended in RPMI‐1640 medium (a concentration of 2 × 10^6^ cells/ml) with a supplement of 10% fetal calf serum.

### 2.8 Assay of splenocyte proliferation

ConA and LPS, were used to assess the T and B lymphocyte proliferative responses, respectively (Lorenzo‐Anota et al., 2021). The CCK‐8 assay was used to evaluate the splenocyte proliferation. The concentration of splenocytes proliferation was changed to 1 × 10^5^ cells/well in DMEM complete medium and planted in 96‐well plate (100 μl/well), and then we added 100 μl ConA (5 μg/ml) or LPS (10 μg/ml) to the wells. For the control group, serum‐free PRMI‐1600 medium (100 U/ml penicillin and 100 μg/ml streptomycin) was used. The cells were incubated at 37°C and 5% CO_2_ for 48 h. CCK‐8 solution (10 μl, 5 mg/ml) was added to each well and then continued to incubation for 2 h. Then, a microplate reader (Thermo) was employed to measure the absorbance at 450 nm. The splenocyte proliferation ratio (%) was A test/A normal × 100%.

### 2.9 Assay of NK cell activity

The effector cells and target cells in this test were splenocytes and YAC‐1 cells, respectively, with the ratio of 50:1. Specifically, splenocytes (100 μl, 5 × 10^6^ cells/ml) and YAC‐1 cells (100 μl, 1 × 10^5^ cells/ml) were co‐cultured in 96‐well plates at 37°C in a humidified incubator with 5% CO_2_. 100 μl RPMI‐1640 medium was used as control. After 24 h incubation, we added CCK‐8 reagent (10 μl, 5 mg/ml) each well and further incubated the cells for 2  h at 37°C. The absorbance was measured at 450 nm via a microplate reader (Thermo). NK cell activity was determined based on the following formula:

$$\mathrm{NK}\;\text{cell activity}\;\left(\%\right)=\frac{A_{\text{experimental release}}-A_{\text{target spontaneous}}}{A_{\text{target maximum}}-A_{\text{target spontaneous}}}\times 100\%.$$

### 2.10 Flow cytometry analysis

The splenocytes were adjusted to the concentration of 1 × 10^7^ cells/ml with 1× staining buffer, a total of 100 μl cell suspension was added in the flow cytometer tube. Then, the cells were completely mixed with 10 μl of PE anti‐mouse CD3, FITC anti‐mouse CD4 and APC anti‐mouse CD8 for 30 min on ice and protected from light. Then, cells were washed with 1× staining buffer for three times and resuspended in 1% paraformaldehyde. The percentage of CD3^+^, CD4^+^ and CD8^+^ T lymphocytes was measured by NovoCyte flow cytometers (ACEA).

### 2.11 Hematoxylin and eosin staining

In this study, the hematoxylin and eosin (HE) staining was conducted as previously reported (Nam et al., 2021). Jejunum tissues were extracted and fixed using 4% paraformaldehyde at room temperature overnight. Afterwards, the tissues were cleaned using distilled water, dehydrated via gradient alcohol, and fixed using paraffin. Samples were sliced into 8 μm thick sections for HE staining. Relevant images were then taken through an optical microscope (Leica DM4000) at 100× magnifications with the same light intensity. The morphological structure of intestinal segments (intestinal villus height, mucosal thickness, and crypt depth) were determined by measuring 10 or more different regions across each section.

### 2.12 Peripheral hemogram analysis

The technique of retro‐orbital bleeding was used to collect blood samples into heparin tubes. An automatic blood cell analyzer (Sysmex, XE‐5000) was used for counting white blood cells (WBC), lymphocytes (LYMPH), and neutrophil (NEUT).

### 2.13 Spleen and thymus indexes

Spleen and thymus from the mice were washed with saline (0.9% NaCl), dried with filter paper, and then weighed. The spleen and thymus indexes were calculated using the following formula: Spleen (thymus) index = the Spleen (thymus) mass (g)/body mass of mice (kg) × 100.

### 2.14 Cytokine assays

Blood samples were taken from the orbital sinus and centrifuged at 1000 *g* for 10 min at 4°C to obtain serum. The cytokine (TNF‐α and INF‐γ) levels of the serum samples were evaluated by ELISA kits according to the manual provided by manufacturer.

### 2.15 16S rDNA sequencing and microbiota analysis

Fecal pellets were collected and snap‐frozen in liquid N_2_. DNA isolated using a TIANamp Stool DNA kit (Tiangen Biotech Co., Ltd) following the manufacturer's recommendations. Assessment of DNA quality and quantity were carried out by calculating their absorbance (A260 and 280 nm) using a NanoDrop 2000 spectrophotometer (Thermo Scientific). To amplify the V3–V4 regions of bacterial 16S rRNA gene segments, the primers 338F/806R were used (Wei et al., 2021). The sequencing were performed on the Illumina MiSeq platform. The analysis of sequencing was based on QIIME 2 (v.2018.6). The α‐ and β‐diversity analysis, the taxonomic composition and Venn diagrams were obtained by R (v.4.0.2).

### 2.16 Statistical analysis

The SPSS 22.0 was employed to analyze the data. Mean and standard deviation (SD) was used to present the data. Basic data are analyzed using *t*‐test (two group comparisons) or one‐way ANOVA (three or more groups of comparisons). *p* < .05 was used to indicate the significant difference.

## 3 RESULTS

### 3.1 Appraisal of the immunosuppressed mouse model

The changes of body weight and status of the mice (before and after the injection of Cyp) were recorded to appraisae if the immunosuppressed model was well‐established. Prior to the injection of Cyp, all mice had shiny hair, bright eyes, and quick movements. However, since the injection of Cyp, the mice became weak with dull and sparse fur, and presented a significant decrease of body weight (Figure 1b). A similar decreasing trend was also observed regarding the spleen and thymus index for mice in the M group when compared with the normal control group (*p* < .05) (Figure 1c,d). The above results indicated the success of the immunosuppressed mouse model.

### 3.2 Effect of PF40 on the indexes of thymus and spleen

The indexes of thymus and spleen were obviously lower in the Cyp‐treated group than in the control group (Figure 1c,d). The spleen index of mice treated with PF40 (all three dosages) was significantly higher than that of the Cyp‐treated group (*p* < .05). For thymus index, it was significantly increased by PF40‐treatment (dosage of 400 mg/kg/day) (*p* < .01) when compared with the Cyp‐treated group. The findings indicated that PF40 could reverse the Cyp‐induced atrophy of immune organs.

### 3.3 Effect of PF40 on blood index

The contents of WBC, LYMPH, and NEUT in different groups of mice were measured (Table 1). A significant reduction in the contents of WBC and LYMPH, and a significant increasing of the NEUT level were observed in the Cyp‐treated group when compared with the control group (*p* < .01). PF40 (400 mg/kg/day) treatment significantly alleviated the changes on Blood index caused by Cyp (*p* < .05 or *p* < .01), which indicated that the PF40 could effectively relieve the change of WBC, LYMPH, and NEUT level induced by Cyp and improve body immunity.

**TABLE 1:** Effect of PF40 on WBC, LYMPH, and NEUT in Cyp‐induced immunosuppressed mice

Groups	WBC (10^9^/L)	NEUT (%)	LYMPH (%)
Control	6.58 ± 0.50	22.75 ± 5.90	67.12 ± 5.98
Cyp	4.48 ± 0.55**	37.07 ± 4.85**	52.00 ± 4.09**
PF40‐L	4.87 ± 0.39	36.10 ± 6.39	55.08 ± 7.54
PF40‐M	5.75 ± 0.52^#^	31.43 ± 2.79^##^	56.78 ± 6.52
PF40‐H	5.85 ± 1.03^#^	33.98 ± 4.72^##^	59.45 ± 3.93^##^

Compared with control group: **p < .01; compared with the Cyp group: #*p* < .05, ##*p* < .01.

### 3.4 Effect of PF40 on carbon clearance rate and phagocytic index

**FIGURE 2:**
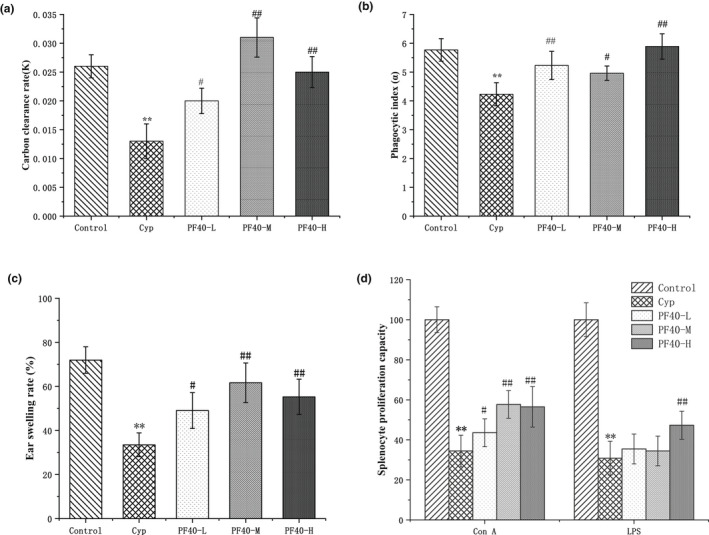
Effects of PF40 on the cellular immunity in CyP‐treated immunosuppression mice (means ± SD, *n* = 10). (a) PF40 increased the rate of carbon clearance of the macrophages. (b) PF40 increases the phagocytic index of the macrophages. (c) Ear swelling rate in DTH reaction; (d) ConA‐ and LPS‐induced splenocyte proliferation. Compared with the control group: **p < .01; compared with the model group: #*p* < .05, ##*p* < .01.

As shown in Figure 2a,b the carbon clearance rate and phagocytic index of the Cyp‐treated group were lower than the normal control group (*p* < .01). The carbon clearance rate in PF40‐treated groups, was significantly higher than that of the model group (*p* < .05). When compared with the model group, a significant increase in the phagocytic index was observed in the PF40 treatments (all three dosages) (*p* < .05). This data implied that PF40 may have the function of activating macrophages.

### 3.5 Effect of PF40 on DTH reactions

The DNFB‐induced delayed type hypersensitivity (DTH) reaction is presented in Figure 2c. Ear swelling rate in model group was obviously lower than the normal control group (*p* < .01). While the administration of PF40 (200 and 400 mg/kg/day) could effectively ameliorated the DTH reaction compared with the model group (*p* < .01). These findings suggested that PF40 strengthened the T‐lymphocyte‐mediated immune response in mice.

### 3.6 Effect of PF40 on serum hemolysin formation

Results in Table 2 illustrated the effect of PF40 on serum hemolysin formation. The level of serum hemolysin in Cyp‐treated mice was significantly lower than that of the control group (*p* < .01). Serum hemolysin formations in the PF40 (400 mg/kg/day) groups increased significantly when compared with the mice in model group. However, no statistically significant difference was observed for PF40 groups at 100 and 200 mg/kg/day compared with Cyp‐treated group (*p* > .05). This data implied that PF40 has a weak effect on humoral immunity.

**TABLE 2:** Effects of PF40 on serum hemolysin level and NK cell activity in Cyp‐induced immunosuppressed mice

Groups	Serum hemolysin (HC50)	NK cell activity (%)
Control	120.54 ± 28.26	30.49 ± 5.03
Cyp	44.81 ± 14.55**	21.70 ± 4.72**
PF40‐L	54.12 ± 15.46	26.14 ± 5.99#
PF40‐M	57.46 ± 19.76	28.26 ± 5.70##
PF40‐H	76.79 ± 23.80##	31.47 ± 6.22##

Compared with control group: **p < .01; compared with the Cyp group: #*p* < .05, ##*p* < .01.

### 3.7 Effect of PF40 on NK cells activity

The YAC‐1 cells were regarded as target cells in this study to assess the activity of NK cells. The activity of NK cells in Cyp‐treated groups was significantly lower than those in the normal control group (*p* < .01) (Table 2), which further indicated that the immunosuppressed mice model was well‐established. However, the activity of NK cells in PF40‐treated groups, regardless of the dosage, was significantly increased (*p* < .05). The above data indicated that PF40 could promote the recovery of the NK cell activity in Cyp‐treated mice.

### 3.8 Effect of PF40 on the proliferation of spleen lymphocytes

Lymphocytes were separated from the spleen of mice in each group, and were treated with ConA or LPS, respectively, to stimulate the proliferation of T and B lymphocytes. In Cyp‐treated group, the response of splenocyte proliferative to ConA or LPS was significantly weaker than that of the normal control group (*p* < .01) (Figure 2d). However, PF40 treatment, regardless of the dosage, could effectively restore the splenocyte proliferative response to ConA (*p* < .05). Meanwhile, mice in the 400 mg/kg/day PF40‐treated group (but not the 100 and 200 mg/kg/day PF40‐treated group), had much stronger splenocyte proliferative responses to LPS than those in the Cyp‐treated group (*p*  <  .01). The result indicated that PF40 could restore Cyp‐treated T‐cell proliferation suppression in mice. However, PF40 has a weak effect on the proliferation of B lymphocytes.

### 3.9 Effect of PF40 on splenic T‐lymphocyte subsets

**FIGURE 3:**
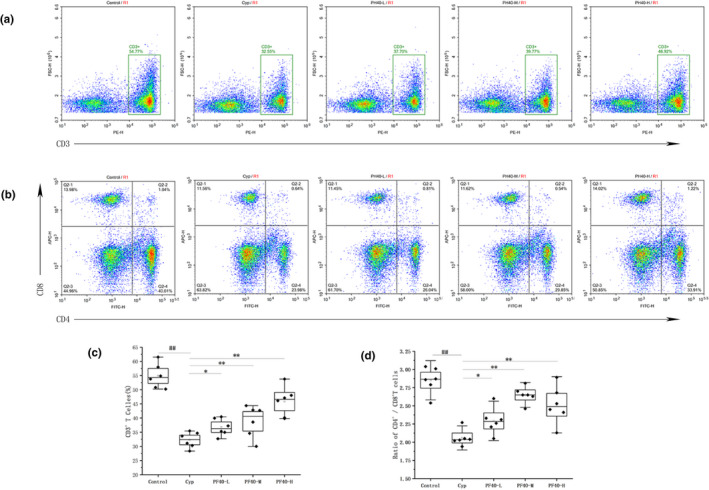
Effect of PF40 on splenic T‐lymphocyte subsets in Cyp‐induced immunosuppression mice (mean ± SD, *n* = 6). (a) the proportion of splenic CD3^+^ T cells was detected by flow cytometry. (b) the proportion of splenic CD4^+^ and CD8^+^ T cells was detected by flow cytometry. (c) the expression of CD3^+^ T lymphocyte. (d) the ratio of splenic CD4^+^/CD8^+^ T cells. **p < .01 versus the control group; #*p* < .05, ##*p* < .01 versus the Cyp‐treated group.

The effect of PF40 on the lymphocyte subsets of Cyp‐induced immunosuppressed mice was evaluated by the flow cytometry. The number of CD3^+^ T lymphocytes in spleens from the Cyp‐treated group was significantly decreased compared with that in the normal control group (*p* < .05), whereas the amount in the PF40 treatment (100, 200 and 400 mg/kg/day) groups was significantly increased compared with that in the model group (*p* < .05; Figure 3). The ratio of CD4^+^/CD8^+^ T lymphocytes was lower in the model mice than those in the normal control (*p* < .01). The ratio of CD4^+^/CD8^+^ T lymphocytes was significantly higher in the PF40 treatment groups (200, 400 mg/kg/day) compared with the model mice (*p*  <  .01). It indicated that PF40 could help regulate the impaired immune system via up‐regulating T‐lymphocyte subsets.

### 3.10 Effect of PF40 on the morphology of jejunum

**FIGURE 4:**
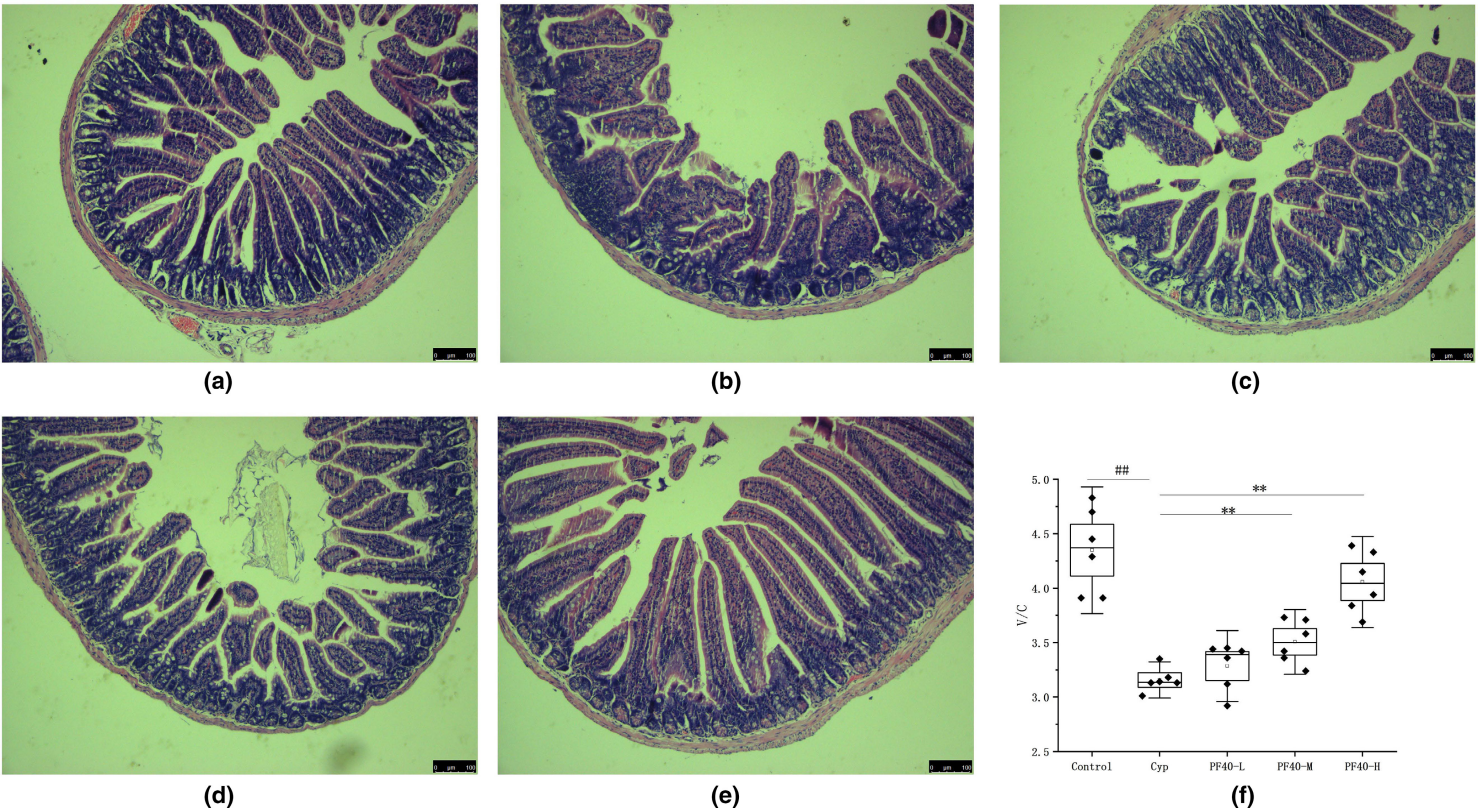
Effects of PF40 on the morphology of jejunum in Cyp‐treated mice (mean ± SD, *n* = 6). Histopathology observation of jejunum stained with H&E (a–e represent the normal control group, Cyp‐treated group, PF40‐L group, PF40‐M group, and PF40‐H group, respectively); (f) the ratio of villus length to crypt depth (V/C). ** *p* < .01 versus the control group; #*p* < .05, ##*p* < .01 versus the Cyp‐treated group.

Previous paper has reported that the high dose Cyp treatment can destroy the gastrointestinal mucosal barrier. To explore the protective effect of PF40 on intestinal mucosa damage, H&E staining was performed. The model group showed shortening of Jejunum villi and destruction of epithelial cell layer (Figure 4). The villus height and crypt depth were significantly decreased in Cyp‐treated group compared with the normal control group (*p* < .01), and those in the PF40 treatment group were significantly improved compared with the model group (*p*  < .05).

### 3.11 Effect of PF40 on the levels of cytokines

**FIGURE 5:**
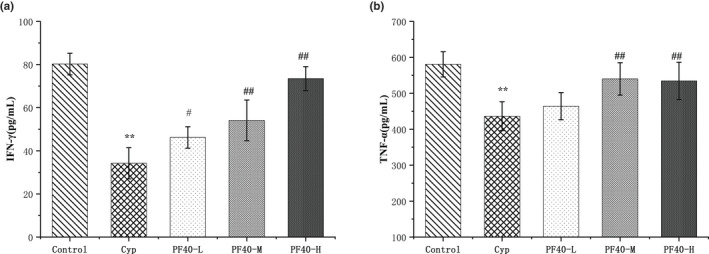
Effects of PF40 on the levels of serum TNF‐α and IFN‐γ in CyP‐treated immunosuppression mice (means ± SD, *n* = 10). (a) PF40 increases the serum TNF‐α level; (b) PF40 increases the serum IFN‐γ level. Compared with the normal control group: **p < .01; compared with the model group: #*p* < .05, ##*p* < .01.

The ELISA protocol was used to evaluate the effects of PF40 on the levels of IFN‐γ and TNF‐α in the serum (Figure 5). The levels of IFN‐γ and TNF‐α in the Cyp group were significantly lower than those of the normal control group (*p* < .05). When compared with the Cyp group, the TNF‐α levels in the PF40‐M (200 mg/kg/day) and PF40‐H (400 mg/kg/day) group were significantly elevated (*p* < .01), and IFN‐γ levels in the PF40‐treated groups (all three dosages) were also significantly increased (*p* < .05 or *p* < .01).

### 3.12 Effects of PF40 on gut microbiota

**FIGURE 6:**
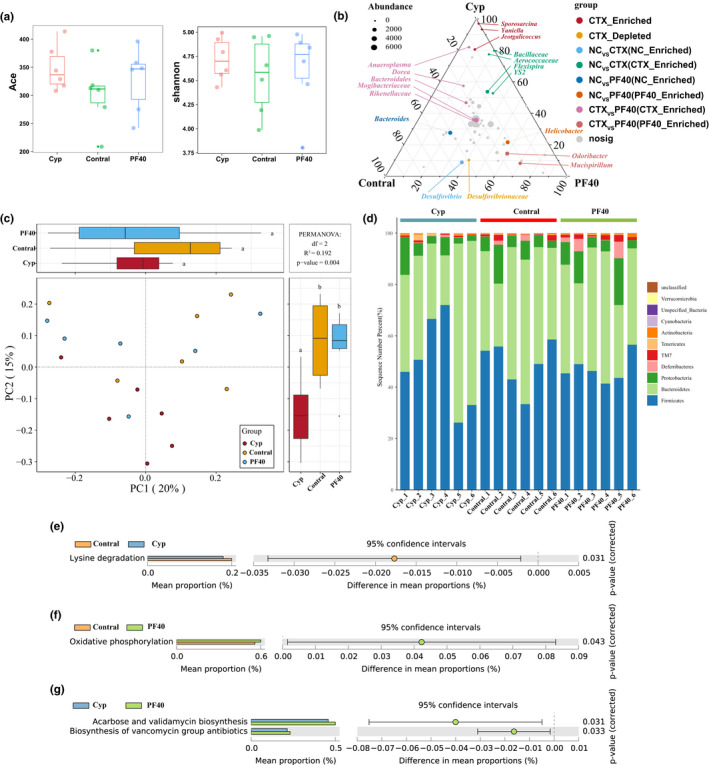
PF40 modulated the composition and function of gut microbiota in different groups (6 per group). (a) Ace index and Shannon index of intestinal microorganisms in different groups. (b) Ternary plot showing compartments specifying enriched and depleted microorganism for microbial community composition. (c) Principal component analysis groups (PCA). PC1 and PC2 represent the two most important factors affecting the layout of microorganisms. (d) Microbial community stack column plot. (e–g) differentially abundant KEGG pathways between the Contral and Cyp, Contral and PF40, Cyp and PF40 groups. All pathways with a *p* < .05 are shown.

We obtained 742,599 high‐quality 16S rRNA gene data for 18 samples. After filtering and clustering with 97% similarity, each sample obtained 325 ± 52 OTUs. The intestinal microbial composition of different treatment groups is shown in Figure 6d. The Ace and Shannon indexes were used to evaluate the beta diversity of the gut microbiota in different treatment groups (Figure 6a). The results showed that the ace and Shannon indexes of different treatment groups were not significantly different (*p* > .05). We used principal co‐ordinate analysis to determine the difference in beta diversity between different treatment groups (Figure 6c). We found that there were significant differences in the PC2 direction of different treatment groups. Compared with the Cyp group, the PC2 value of the PF40 intervention group was significantly increased (*p* < .01). This result suggests that PF40 can increase the beta diversity of the gut microbial composition of immunosuppressed mice. We performed a ternary graph analysis between the control group, the Cyp group and the PF40 group to determine specific enriched and depleted bacterial genera (Figure 6b). The results showed that *Sporosarcina*, *Yaniella*, and *Jeotgalicoccu*s increased significantly in the Cypgroup (*p* < .05), while *Desulfovibrionaceae* decreased significantly (*p* < .05). In contrast, *Odoribacter* and *Mucispirillum* increased significantly after PF40 intervention treatment.

To predict the function of intestinal microbes, we matched these data with the sequence data annotated by KEGG (Figure 6e–g). The functional difference analysis of KEGG showed that the function of lysine degradation category was significantly reduced in the Cyp group compared with the control group (*p* < .05), while the function of oxidative phosphorylation category in the PF40 group was significantly increased (*p* < .05). Compared with the Cyp group, the acarbose and validamycin biosynthesis function was significantly increased after PF40 intervention (*p* < .05), and the function of Biosynthesis of Vancomycin Group Antibiotics was also significantly increased (*p* < .05).

**FIGURE 7:**
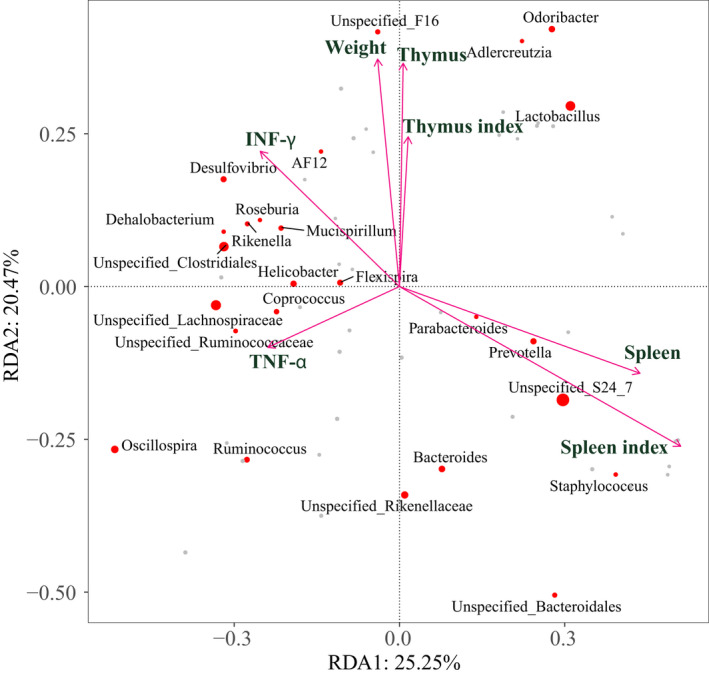
Redundancy analysis identified seven selected physiological parameters for shaping microbial community.

We correlated the data of intestinal microbial composition with serum biochemical indicators (TNF‐α and INF‐γ) and immune organ indexes (body weight, spleen weight, spleen index, thymus weight and thymus index) (Figure 7). Redundant analysis (RDA) was performed to quantify the influence of physiological and biochemical indicators on the composition of the intestinal bacterial community. As demonstrated by the RDA biplot, spleen weight and spleen index are the main factors affecting the composition of the bacterial community. The weight and thymus index of mice have a weak influence on the composition of the bacterial community. *Parabacteroides*, *Prevotella*, *Staphylococcus*, and *Bacteroides* are positively correlated with spleen weight and spleen index, and negatively correlated with TNF‐α and INF‐γ levels.

## 4 DISCUSSION

Cyp is a commonly used antitumor drug, but it has a strong immunosuppressive effect. Cyp can destroy the structure of DNA, restrain the lymphocytes proliferation, and inhibit the cellular and humoral immune response (Ayza et al., 2020; Bracci et al., 2014). Cyp‐treated mice is a commonly used immunosuppressed mice model for screening immunomodulatory drugs (Daillère et al., 2016; Lv et al., 2021). In this study, we replicated this model by injection Cyp (50 mg/kg for two consecutive days) intraperitoneally to verify the immune enhancement effect of PF40. As expected, mice showed obvious immunosuppression after the Cyp treatment, with decreased immune organ indexes, phagocytic ability of macrophages and lymphocyte proliferation. These significant differences in immune indexes between the Cyp treatment group and the normal group suggested that the immunosuppressive mice model is well‐established in this study.

Spleen and thymus are two important lymphoid organs for differentiating and maturing of immunological cells. The functional status of immune organs are closely associated with the host's immune function (Li et al., 2020; Liu et al., 2019; Niu, Cao, et al., 2020). Their relative weights are considered to be the key and intuitive indicators of nonspecific immunity (Xie et al., 2015). When the immune function of the body is reduced, the spleen and thymus will shrink. Spleen index and thymus index have been commonly used as two conventional indicators for the entire immune status assessment of the organism. This study indicated that PF40 treatment could increased the spleen and thymus indices in immunosuppressive mice. The results indicated that PF40 could promote the development of immune organs.

Mononuclear macrophages are essential effector cells in nonspecific immune responses and play a crucial role in innate and acquired immunity regulation (Dai et al., 2021). The rate of carbon clearance reflects the phagocytic ability of mononuclear phagocytes. Findings from this study showed that PF40 can significantly increase the carbon clearance rate and phagocytic index as well as the phagocytic ability of mononuclear macrophages in Cyp‐induced immunosuppressive mice, which suggested that PF40 could initiate nonspecific immunity in an immunosuppressive condition. This study finding was supported by a previous study (Niu, Dong, et al., 2020), which found that *Malus halliana* flower polysaccharide can enhance the ability of carbon clearance and phagocytosis of mononuclear phagocytes in mice.

Delayed type hypersensitivity, as the IV type of hypersensitivity reaction, is a kind of cytoimmunity that mediated by T lymphocytes, which plays an important role in evaluating T‐lymphocytes‐mediated immune responses (Liu et al., 2019). In this study, DNFB was used to activate ear DTH reaction, and it was found that the rate of ear swelling in mice was increased after the PF40 treatment. It indicated that the PF40 can enhance the cell‐mediated immune via counteracting the inhibitory effect of Cyp on the DTH reaction.

Sheep red blood cells are a powerful antigen to mice, and immunization with SRBC can lead to the production of hemolysin antibody in the serum (He et al., 2016). The level of serum hemolysin as an important indicator of humoral immunity can reflect the ability of B cells to secrete hemolysin after contact with specific antigens (Jie et al., 2015). According to the current study findings, we can preliminarily conclude that the B cells might be less sensitive to PF40 as only the high dose of PF40 can significantly increase the level of serum hemolysin.

The proliferation ability of lymphocytes is the most direct indicator for evaluating the function of T lymphocytes (Guo et al., 2021). T lymphocytes are primarily responsible for cellular immunity, which could reject the foreign tissue, devastate cancer cells, and inhibit the proliferation of harmful cells. B lymphocytes are mainly responsible for humoral immunity, and they can produce a variety of antibodies once activated by antigens. ConA and LPS could induce T and B lymphocytes proliferation, respectively. In this study, the study findings indicated that cyclophosphamide can significantly inhibit the proliferation of spleen lymphocytes. PF40 could ameliorate Cyp‐induced T‐lymphocyte proliferation suppression in mice. Interestingly, low dose of PF40 had only weak effects on the proliferation activity of B lymphocytes; while a significant enhancement regarding the proliferation activity of B lymphocytes was identified in the group of high‐dose PF40. It means that PF40 has less effect on B lymphocytes. This finding was also supported by the result of the serum hemolysin test.

NK cells, as a subset of innate lymphoid cells, play a crucial role in the surveillance of immune functions, which can regulate the dendritic cells or T cells to achieve the purpose of modulating adaptive immune responses (Li et al., 2018; Park et al., 2021). The NK cell activity, as an important indicator, could judge the immunity of organism. In this study, the cytotoxic activity of splenic lymphocytes was measured against NK cell‐sensitive cells (YAC‐1). The results indicated that the Cyp could significantly decrease the mice's splenic NK cell activity, while PF40 could promote the recovery of splenic NK cell activity.

According to the modern pharmacological research findings, the amount of T lymphocytes and ratio of CD4^+^/CD8^+^ can reflect the body's immune function status and the severity of a disease (Ynga‐Durand et al., 2021; Zhuang et al., 2021). CD3^+^ T cell is the general term for all mature T lymphocytes, and reflects the strength of cellular immunity directly (Guo et al., 2017). CD4^+^ cells indicate the helper T cells, which can secrete a variety of lymphokines and help maintain immune homeostasis. CD8^+^ cells indicate suppressor T cells. The decreased ratio of CD4^+^/CD8^+^ T indicates a downregulation of immune response, which means that the body's immune system is relatively weak. Therefore, the ratio of T lymphocytes can be used as an indicator for evaluating the function of immune system. Consistent with previous studies (Han et al., 2020), this current study also showed that the amount of CD3^+^ T cells and ratio of CD4^+^ / CD8^+^ were decreased in Cyp‐treated mice. Besides, the proportions of CD3^+^ and the CD4^+^/CD8^+^ ratio were obviously improved after the PF40 treatment. Hence, it is conceivable that PF40 may improve the immune function of immunosuppressed mice by affecting the levels of CD4^+^ and CD8^+^ T cells.

The intestine not only has diverse functions, such as digestion, absorption, secretion but also is the main component of the body's mucosal immune system (Ghosh et al., 2021; Kurashima et al., 2013). The intestinal mucosal immune system deals with the stimulation of tens of millions of antigens including pathogens, food proteins, and microbiome everyday (Heller & Duchmann, 2003; Kurashima et al., 2013). It is the body's first line of defense against intestinal pathogen infection. Moreover, it plays an important role in the establishment and maintenance of mucosal homeostasis between the host and the external environment. The normality of the intestinal mucosal morphology and structure reflects the level of intestinal mucosal immunity, which is directly related to the function of the intestinal mucosal immune system (Olivares‐Villagomez & Van Kaer, 2018; Shi et al., 2017). Our research results show that PF40 can promote the growth of jejunal villi, accelerate the maturation of intestinal epithelial cells, increase the ratio of villus length to crypt depth, enhance intestinal absorption, inCyp‐induced immunosuppressive mice.

Cytokine is a highly active protein, secreted by the activated T cells, and plays an important role in the immune response (Morris et al., 2018). Disturbance of cytokine levels may lead to the occurrence and development of diseases. TNF‐α is mainly secreted by active macrophages and monocytes, which is a crucial cytokine for immune response. IFN‐γ promotes the activation of antigen presenting cells. TNF‐α and IFN‐γ are two important cytokines for the development of immune cells; the decrease in the content of TNF‐α and IFN‐γ may cause disorders of lymphokines and lead to a decline in immune function (Chen, Jiao, et al., 2016; Jung et al., 2019; Yu et al., 2018). The results in this study showed that Cyp significantly decreased the content of TNF‐α and IFN‐γ, while PF40 inhibited the Cyp‐induced reduction and increased the immune function of mice.

Hundreds of microorganisms live in the intestines of mammals. The microbiota plays an important role in human health and disease, and there is evidence that the gut microbiota plays a key role in shaping the immune system. Plant polysaccharides could improve immune function by regulating the intestinal microbial ecology. In this study, we studied the effect of PF40 on the composition of the gut microbiota in mice treated with Cyp. The result showed that PF40 increased Beta diversity compared with Cyp group. The microbial diversity in the intestine is the basis for the microbiota to play an immunomodulatory role. In this study, we found that the abundance of *Sporosarcina*, *Yaniella*, and *Jeotgalicoccus* significantly increased in the Cyp group. According to previous reports, *Sporosarcina* is related to the intestinal immune inflammatory response (Wang et al., 2020), and *Yanilela* is negatively correlated with the liver glycogen level of the body (Xiao et al., 2020). The increase of *Yaniella* level will inhibit the synthesis of liver glycogen, thereby interfering with the body's energy metabolism. In contrast, PF40 treatment increased the relative abundance of *Odoribacter* and *Mucispirillum. Odoribacter* could stimulate the production of IL‐6 and IL‐1β and the expansion of Th17 cells to confer resistance to colitis and colon cancer (Foegeding & Byndloss, 2021). *Odoribacter* is positively correlated with the content of isobutyric acid in fecal short‐chain fatty acids (Granado‐Serrano et al., 2019). *Mucispirillum* can prevent colitis induced by *Salmonella typhimurium* by interfering with pathogen invasion (Herp et al., 2021). Notably, the abundance of *Helicobacter* in the PF40 group was significantly increased compared with control group. It indicated that PF40 could not inhibit the increase in the abundance of *Helicobacter* in the intestine induced by Cyp. *Helicobacter* may be a risk factor for asymptomatic small intestinal ulcers (Fujimori, 2021). The results of the RDA biplot analysis showed that PF40 intervention can increase the relative abundance of *Parabacteroides*, *Prevotella*, *Staphylococcus*, and *Bacteroides*, which are positively correlated with immune status. In general, PF40 was beneficial to increase the abundance of beneficial bacteria related to inflammation and energy metabolism in the intestine. However, the disordered state of the intestinal flora structure caused by Cyp was still not optimistic.

Results of changes in Spleen indexes, macrophage phagocytosis, splenocyte proliferation, NK cell activity, DTH, and flow cytometry aAnalysis indicated that all dosage groups of PF40 exhibited therapeutic action to a certain extent, when compared with M group. The efficacies of the PF40‐H treatment group and the PF40‐M treatment group were better than that of the PF40‐L treatment group. Therefore, the therapeutic dose of PF40 recommended for Cyp‐induced immunosuppressed mice is 200–400 mg/kg b.w., while 100 mg/kg b.w. can be used for prevention or consolidating its curative effect. The results provide predictive evidence that PF40 might be a promising natural functional food for reducing chemotherapy‐induced immunosuppression.

## 5 CONCLUSIONS

Given together, this current study concluded that the polysaccharides (PF40) from *P. heterophylla* can enhance the immune function, improve atrophy of liver and spleen, reduce histopathological changes of Jejunum tissue, increase the number of leukocytes. Besides, PF40 can enhance the cell‐mediated immunity via improvements in macrophage phagocytosis, splenocyte proliferation, NK cell activity and DTH, as well as improve humoral immunity via promoting the formation of serum hemolysin. Meanwhile, PF40 can elevate the proportion of CD3+ and the ratio of CD4+/CD8+ in Cyp‐induced mice. PF40 regulates the intestinal microbiota by restoring the relative abundance of *Odoribacter* and *Mucispirillum* and reducing the relative abundance of *Sporosarcina*, *Yaniella*, and *Jeotgalicoccus* in Cyp‐treated mice. In conclusion, PF40 could be applied as an effective auxiliary immune enhancing agent to restrain Cyp‐induced decrease in immune function. More detailed molecular mechanism of immunomodulatory activity of PF40 is worthy of further explore in future studies.

## CONFLICT OF INTEREST

The authors declare no conflict of interest.

## ETHICS STATEMENT

This study were granted by the Institutional Animal Care and Use Committee at Fujian Academy of Chinese Medical Sciences (no. FJATCM‐IAEC2021007).

## Data Availability

Data utilized to support the findings of this study are available from the corresponding author upon reasonable request.
